# CTT: CNN Meets Transformer for Tracking

**DOI:** 10.3390/s22093210

**Published:** 2022-04-22

**Authors:** Chen Yang, Ximing Zhang, Zongxi Song

**Affiliations:** 1 Xi’an Institute of Optics and Precision Mechanics of CAS, Xi’an 710000, China; yangchen@opt.cn (C.Y.); zhangximing@opt.ac.cn (X.Z.); 2 University of Chinese Academy of Sciences, Beijing 100049, China

**Keywords:** self-attention, tracking, transformer, CNN

## Abstract

Siamese networks are one of the most popular directions in the visual object tracking based on deep learning. In Siamese networks, the feature pyramid network (FPN) and the cross-correlation complete feature fusion and the matching of features extracted from the template and search branch, respectively. However, object tracking should focus on the global and contextual dependencies. Hence, we introduce a delicate residual transformer structure which contains a self-attention mechanism called encoder-decoder into our tracker as the part of neck. Under the encoder-decoder structure, the encoder promotes the interaction between the low-level features extracted from the target and search branch by the CNN to obtain global attention information, while the decoder replaces cross-correlation to send global attention information into the head module. We add a spatial and channel attention component in the target branch, which can further improve the accuracy and robustness of our proposed model for a low price. Finally, we detailly evaluate our tracker CTT on GOT-10k, VOT2019, OTB-100, LaSOT, NfS, UAV123 and TrackingNet benchmarks, and our proposed method obtains competitive results with the state-of-the-art algorithms.

## 1. Introduction

**Figure 1:**
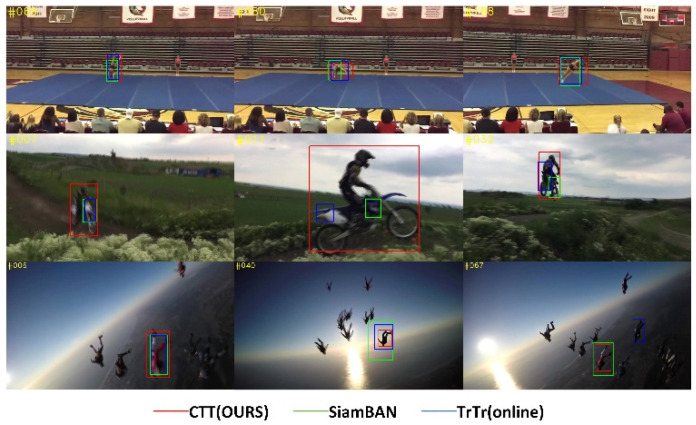
A comparison of our network with the state-of-the-art trackers SiamBAN [7] and TrTr (online) [8]. It can be seen from the visualization results that our tracker is more accurate and robust when facing the object with appearance change, scale change, and occlusion.

Visual tracking is a vital cornerstone of drone application, autonomous driving and video surveillance [1]. The methods based on deep learning have gradually become the dominated methods of the visual object tracking on account of their excellent balance in accuracy and speed. SiamRPN++ [2] and ViT [3] have become milestone works about Siamese network and visual transformer. The advantage of Siamese networks is that the backbone architecture can be shared between the target branch and search branch, and the target branch weights will not be upgraded in the rest frames. The transformer’s competitive strength is neatly illustrated by full fusion of the low-level feature maps so as to gain global attention. The feature fusion module of most Siamese networks completely copies with the SiamRPN++, and then completes the feature matching through cross-correlation. We consider that the inputs of the head module should be feature maps rather than some probability values [4]. Information density is different between language and vision. Languages are human-generated signals that are highly semantic and information-dense. Images, on the contrary, are natural signals with heavy spatial redundancy [5]. The convolution kernel in CNN can easily extract the low-level features, and the encoder-decoder structure in transformer leads itself to well fuse dense information [6]. So, we apply the CNN as backbone and transformer in the feature fusion module to obtain global attention, resulting in more accurate and compact tracking as shown in Figure 1.

The attention mechanism has become a highlight in target detection, but rarely in tracking. For a long time, series of ResNet [9] are set as the backbone to the Siamese trackers without differences between the target branch and search branch. Inspired by SENet [10] and CBAM [11], the spatial and channel attention components are added to the target branch to make its target feature focus on the target itself. Therefore, the main highlights of this paper are redesigning the feature fusion component and making low-level features from backbone more suitable for tracking. Our main contributions are three-fold:

1. We propose a novel stacked self-attention network for object tracking (named CTT), which consists chiefly of the encoder-decoder transformer architecture. It is worth noting that we use CNN with spatial and channel attention for extracting features in order to better adapt to the tracking task, and we use transformer for encoding and decoding them to complete tracking task;
2. We add a spatial and channel attention component in the target branch that can effectively assist the network to be more concerned with identifying objects;
3. We conduct comprehensive experiments on large-scale benchmark datasets including GOT-10k [12], VOT2019 [13], OTB-100 [14], NfS [15], UAV123 [16], and TrackingNet [17], and our tracker achieves competitive results which demonstrate the effectiveness of the proposed method while running in real-time speed.

## 2. Related Works

Tracking is a very common high-level visual task in computer vision. In this section, we will illustrate the visual object tracking and recent developments in the visual transformer. In addition, we will introduce additional knowledge about spatial attention and channel attention.

### 2.1. Visual Tracking

Visual tracking is one of the most active research topics in computer vision, and most of the recent state-of-the-art trackers are Siamese-network based. Tao et al. propose the SINT [18] as the pioneering Siamese network which considers no parameters of fine-tuning during network inferencing. Bertinetto et al. introduce the SiamFC [19] as an end-to-end tracking network which contains the template branch and search branch to accomplish the matching strategy. Li et al. propose the SiamRPN [20] that rely on the large-scale dataset to finish the training process leading to a significant improvement in the metrics of accuracy and robustness. Li et al. also find the way of applying the deeper backbone network (ResNet) when training the whole framework, namely SiamRPN++. The SiamRPN++ has gained the great enhancement in the tracking performance, which proves that the deeper network can be employed to the tracking frameworks. However, the lack of attention mechanisms makes the SiamRPN++ with little regard for the input itself for information interaction during feature learning. The SiamMask [21] uses the mask as the output of regression, and according to the minimum bounding box drawn from the segmented mask to realize the accurate tracking of the object. Yu et al. propose the SiamAttn [22] to obtain a highly effective Siamese attention mechanism, including self-attention and cross-attention, which provides an adaptive approach to implicitly update template characteristics. In this way, the tracker can achieve much better target discrimination during the training process.

### 2.2. Visual Transformer

An attention function can be described as mapping a query and a set of key-value pairs to an output, where the queries, keys, values, and output are all vectors. Without the addition of attention in the network, all features of the image are treated equally. That is to say, these features have no difference in the neural network’s “eyes” and the neural network will not pay much attention to a specific area such as a human.

The encoder-decoder transformer architecture has begun to be widely used in natural language processing (NLP). In the last two years in computer vision, IGPT [23], ViT, etc., have successfully applied transformer to image classification, and achieved the state-of-the-art results. TTSR [24], IPT [25], etc., also have achieved success in the low-level visual tasks such as image super resolution and noise reduction, etc. DETR, TSP [26], VisTR [27], and Max-Deeplab [28] have refreshed the evaluation results of object detection and segmentation benchmarks. Trackformer [29], Transtrack [30], and TransCenter [31] are the latest achievements in the field of multi-target tracking. However, the speed of the visual transformer trackers is much lower than that of CNN-based methods. The transformer we redesigned includes six encoders and five decoders. In particular, in order to reduce model parameters to make the network more suitable for the real-time requirements of tracking, there is only one Feed-Forward network (FFN) layer in the decoder. By the way, additive attention is not employed in calculating the similarity of (K, V), but scaled dot-product attention. Scaled dot-product makes the attention operation in transformer become pure matrix multiplication which is convenient for GPU to speed up the training process.

### 2.3. Spatial Attention and Channel Attention

There are many existing attention-based correlation filters, including SDCF [32], SRDCF [33], and DSAR-CF [34]. They all improve the accuracy and robustness of correlation filter-based object tracker by adding regularized items. While in the CNN-based networks, the essence of the attention mechanism is to promote CNN to extract useful information from images with heavy spatial redundancy. Common CNN-based attention in computer vision is mainly divided into three types: the spatial attention (SA), the channel attention (CA), and the spatial and channel mixed attention (SA&CA). Google DeepMind proposes a spatial transformer network (STN) [35]. It is a space-based attention model by adding a deformable attention branch which can learn the input image to complete the preprocessing operation. For 2D image as CNN’s input, one dimension is the scale space of image, that is, length and width, and the other dimension is channel. SENet is the champion network of the 2017 ImageNet classification competition. It calculates the importance of each feature channel, and then adaptively enhances or suppresses different channels for different tasks. The CBAM has a cascaded connection between channel attention and spatial attention to obtain the mixed attention. Our attention module uses the spatial attention with channel attention in parallel with the target branch for accelerating attention computation.

## 3. Our Approach

**Figure 2:**
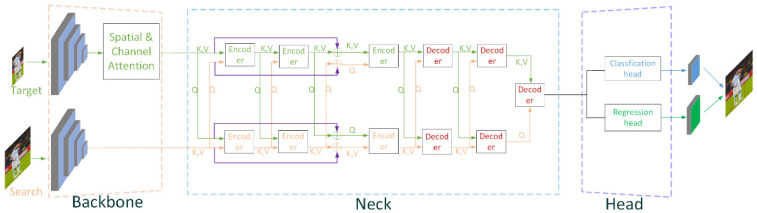
Main framework of CTT: the backbone includes ResNet-50 and attention module (the pink dashed box). The neck has the function of feature fusion (the light blue dashed box). In order to prevent the gradient of the network from disappearing in the process of backpropagation, we use skip connections between encoder and encoder. The head part is used for outputting classification and regression maps (the light purple dashed box).

In this section, we describe the CTT framework building upon our proposed transformer architecture. As shown in Figure 2, there are three modules in CTT: the backbone, the neck, and the head. Among them, the backbone functions as extracting the low-level features from the target and search branch, which uses convolutional network and attention. The neck is applied for encoding the obtained low-level features and decoding them. The head includes classification and regression submodules, respectively generating classification and regression maps. The output classification maps are used to determine whether the object in the search image is a background or an object, and the regression maps are applied to determine the precise location of the object.

### 3.1. Feature Extraction Module

**Figure 3:**
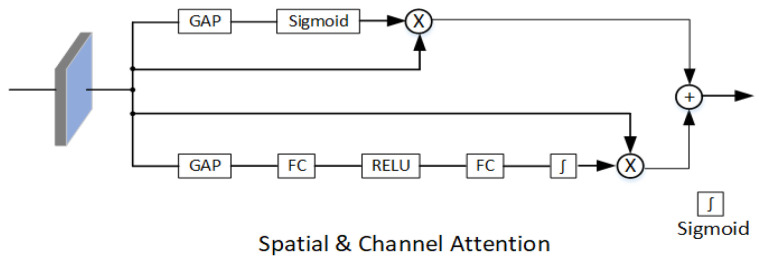
The attention module is composed of spatial attention and channel attention in parallel. Spatial attention only consists of global average pooling (GAP) and activation function sigmoid. Channel attention consists of GAP, fully connection (FC), RELU, and sigmoid.

The backbone of our CTT is modified on the basis of the ResNet-50 pretrained in the ImageNet [36]. In particular, we added a parallel module for the spatial attention and channel attention in the target branch that makes it easier to distinguish selecting the target from similar objects in the background. See Figure 3 for details. Considering that the ResNet-50 has four residual blocks, generally in Siamese networks for tracking, the features after block 2, 3, 4 are extracted separately and then fused. We selectively use the features after the third block to reduce the amount of backbone parameters.

The traditional self-attention mechanism relies on the input itself for information interaction in spatial domain and ignores the rich contextual information between the neighborhood pixels in the image. To alleviate this problem, we insert CNN into the network to fully explore the contextual information between the contextual information, and then add spatial attention and channel attention to the target branch. In this way, the backbone not only can improve the self-attention learning effect, but also improve the expression ability of output features.

### 3.2. Feature Fusion Module

**Figure 4:**
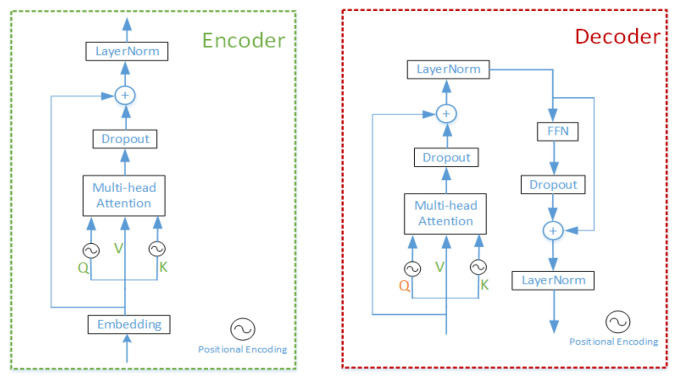
The encoder and decoder in CTT for object tracking. The encoder has less one skip FFN connection than the decoder, which can not only significantly reduce the model size, but also effectively prevent over-fitting caused by dense network parameters in FFN.

Feature fusion module is composed of the simplified encoder-decoder transformer structure. Different from the ViT, DETR, and TrTr, the fusion module consists of six encoders and five decoders, where only decoders use the Feed-Forward network. In our proposed transformer architecture, we use the skip connections such as ResNet-50 to avoid the gradient vanishing effectively. As shown in Figure 4, encoder has a simplified structure compared with decoder. The encoder-decoder structure of original transformer in NLP is symmetrical, because the translated word needs to correspond to the original text one by one in the text translation task. However, in the visual task, we think that the encoder is used to fuse the input feature sequences, so that this information can interact with each other. The decoder is mainly utilized for deciphering the information that has been exchanged in the image and output it to the head module. The multi-head attention in encoder can fulfill the task of information interaction, so we redesigned the encoder-decoder structure by abandoning the FFN layer and the corresponding Layer Normalization (LN) layer in the encoder; specific ablation experiments can be seen in Section 5.

As shown in Figure 2, we mix a pair of feature vectors *Q* and *K* in the target branch with *V* in the search branch, and send them into the encoder in the target branch. We always put *K* and *V* together; *Q* in the target branch and *K* in the search branch will be multiplied by *V* in the target branch after the similarity calculation, so that the search branch and the target branch can have a better interaction of global attention interaction. We do something similar with the search branch.

**Figure 5:**
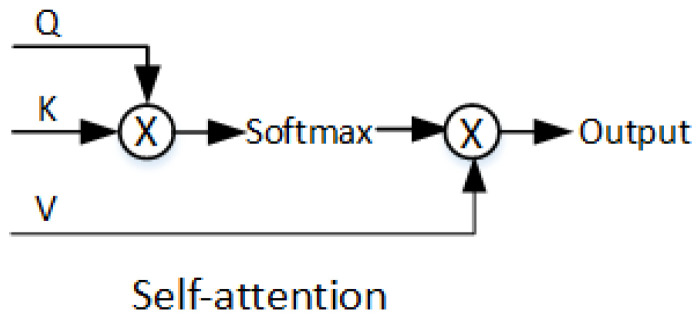
Query-Key-Value attention mechanism.

Query-Key-Value attention is the most important core component in the encoder and the decoder; the calculation process is given as follows:

Assume that the input of encoder is f and becomes $\overset{⌢}{f}$ after embedding, then multiply them by different initial weights to obtain $Q^{i}$, $K^{i}$, $V^{i}$, where $Q^{i}$ and $K^{i}$ have position encoding, on the contrary, $V^{i}$ has not. t or s in the variable subscript indicates that it is on the target branch or search branch. The variables of $Q^{i}$, $K^{i}$, and $V^{i}$ after multi-head attention are referred to as *MHA* which is shown in Figure 5 (we have just drawn up a single-head attention for an example).

(1) $$\{\begin{array}{c} MHA_{t}=Soft\max(Q_{s}^{i}⊙K_{t}^{i})*V_{t}^{i}\\ MHA_{s}=Soft\max(Q_{t}^{i}⊙K_{s}^{i})*V_{s}^{i} \end{array}$$

So, the result of encoder is:

(2) $$\{\begin{array}{c} En_{t}=LN(V_{t}+dropout(MHA_{t}))\\ En_{s}=LN(V_{s}+dropout(MHA_{s})) \end{array}$$

where $Q^{i}⊙K^{i}=Q^{i}*K^{i}/T$, T is a hyperparameter whose value is equal to the square root of FFN hidden layer, ∗ represents dot product, different i means different head. LN represents Layer Normalization.

The decoder has one Feed-Forward network and *LN* layer more than the encoder; assume that the inputs of the decoder are $\bar{Q^{i}}$, $\bar{K^{i}}$, $\bar{V^{i}}$; the variables of $\bar{Q^{i}}$, $\bar{K^{i}}$, $\bar{V^{i}}$ after multi-head attention are referred to as $\bar{MHA}$. Similar to the encoder, $\bar{MHA}$ can be obtained as follows:

(3) $$\{\begin{array}{c} \bar{MHA_{t}}=Soft\max(\bar{Q_{s}^{i}}⊙\bar{K_{t}^{i}})*\bar{V_{t}^{i}}\\ \bar{MHA_{s}}=Soft\max(\bar{Q_{t}^{i}}⊙\bar{K_{s}^{i}})*\bar{V_{s}^{i}} \end{array}$$

then, the output of the decoder can be calculated as follows:

(4) $$\begin{array}{l} \{\begin{array}{c} De_{t}{}^{temp}=LN(\bar{V_{t}}+dropout(\bar{MHA_{t}}))\\ De_{s}{}^{temp}=LN(\bar{V_{s}}+dropout(\bar{MHA_{s}}))\\ De_{t}=LN(De_{t}{}^{temp}+dropout(FFN(De_{t}{}^{temp})))\\ De_{s}=LN(De_{s}{}^{temp}+dropout(FFN(De_{s}{}^{temp}))) \end{array} \end{array}$$

### 3.3. Skip Connections

As you can see in Figure 4, encoder itself contains a residual Query-Key-Value struc-ture to alleviate the vanishing gradient problem. However, when the number of encoders is increasing, the vanishing gradient problem among encoders is rarely mentioned. Hence, we add skip connections between encoder and encoder that can be seen in Figure 2, as shown in purple colors; specific ablation experiments can be found in Section 5 of Chapter 5.

Each skip connection unit in our network can be expressed in a general form:

(5) $$a^{l+1}=a^{l}+F(a^{l})$$

where $a^{l}$ and $a^{l+1}$ are input and output of l−th unit, and F is a residual function. Recursively, we will have:

(6) $$\{\begin{array}{c} a^{l+2}=a^{l+1}+F(a^{l+1})=a^{l}+F(a^{l+1})+F(a^{l})\\ \ldots \\ a^{L}=a^{l}+\sum_{i=l}^{L-1}F(a^{i}),L>l \end{array}$$

Denoting the loss function as ϕ, from the chain rule of backpropagation, we have:

(7) $$\begin{array}{l} \frac{\partial \phi }{\partial a^{l}}=\frac{\partial \phi }{\partial a^{L}}\frac{\partial a^{L}}{\partial a^{l}}=\frac{\partial \phi }{\partial a^{L}}\frac{\partial }{\partial a^{l}}(a^{l}+\sum_{i=l}^{L-1}F(a^{i}))\\ =\frac{\partial \phi }{\partial a^{L}}(1+\frac{\partial }{\partial a^{l}}\sum_{i=l}^{L-1}F(a^{i})) \end{array}$$

Equations (6) and (7) suggest that the signal can propagate directly from any unit to another. In general, the term $\frac{\partial }{\partial a^{l}}\sum_{i=l}^{L-1}F(a^{i})$ cannot be always −1 for all samples. This implies that the gradient of a layer does not vanish even when the weights are arbitrarily small [37].

### 3.4. Loss Function

After the decoder outputs the feature vectors, the head module passes them through the classification and regression heads to generate temporary results of classification and regression. So, the crucial step is how to determine the positive and negative samples. We select the prediction vector which is in the ground truth box as the positive samples. $L_{ce}$ means cross-entropy loss [38].

(8) $$L_{ce}=-\sum_{i=1}^{i=m}\left[y^{i}\ln(p^{i})+(1-y^{i})\ln(1-p^{i})\right]$$

where i means the i−th sample and m is the number of samples, $y^{i}=1$ or $y^{i}=0$ indicates that the i−th sample in the labels is the object or background. $p^{i}$ is the probability that our CTT predicts whether the object is to be tracked.

We use the variant of IOU loss [39], which is called complete IOU (ciou) loss [40], and try to add an $L_{2}$ regular term [41] as penalty, but we observe that the $L_{2}$ regular term will make the network converge not well; finally, we chose the $L_{1}$ regular term.

(9) $$L_{reg}=\sum_{i=1}^{i=m}\left[\lambda_{1}L_{ciou}(p_{b}^{i},b^{i})+\lambda_{2}L_{1}(p_{b}^{i},b^{i})\right]$$

where i means the i−th sample and m is the number of samples. $p_{b}^{i}$ is the prediction box generated from the CTT, and $b^{i}$ is the object’s ground truth in the label. We simply set $\lambda_{1}=2$, $\lambda_{2}=5$ in order to keep every loss in $L_{reg}$ in the training process staying on an order of magnitude.

The total loss $L_{total}$ can be obtained by adding the classification loss $L_{ce}$ and regression loss $L_{reg}$:

(10) $$L_{total}=\lambda_{ce}L_{ce}+L_{reg}$$

As our classification loss and regression loss are calculated on positive samples, and the general classified loss is calculated on all samples, we set the proportion of classified loss and regression loss to 8:1, that is, to keep the balance of classification loss and regression loss.

## 4. Results

In this section, firstly, we describe the implementation details of the proposed approach. Afterward, we compare our model with the competing baseline models on different public datasets.

### 4.1. Implementation Details

We use the ResNet-50 pretrained on the ImageNet as the backbone of our model. The size of the target branch and the search branch are 128×128 and 256×256 for training, respectively. The CTT is trained on the datasets of COCO [42], TrackingNet, LaSOT [43], and GOt-10k. Due to transformer’s poor convergence on the SGD optimizer [44], we apply AdamW [45] with each iteration yield of 1000 training pairs. We train the network on single Nvidia 2080 RTX GPU with the batch size of 8, on a total of 600 epochs with 1000 iterations per epoch. The backbone’s learning rate starts from $2\times 10^{-6}$, and $2\times 10^{-5}$ for other module parameters, then these learning rates decrease by factors of 10 after 300 epochs. Our model is implemented in Python 3.7 using Torch 1.8 based on Ubuntu 20.04 with Intel (R) i9-10980XE CPU 3.000 GHz, 64 G RAM, single Nvidia RTX 2080.

### 4.2. Comparison with the State-of-the-Art

We compared the CTT tracker with the state-of-the-art trackers across six representative benchmarks, and it is worth noting that our hyperparameters are consistent across all benchmark tests, which means no requirement for fine tuning during referencing.

#### 4.2.1. GOT-10k

**Figure 6:**
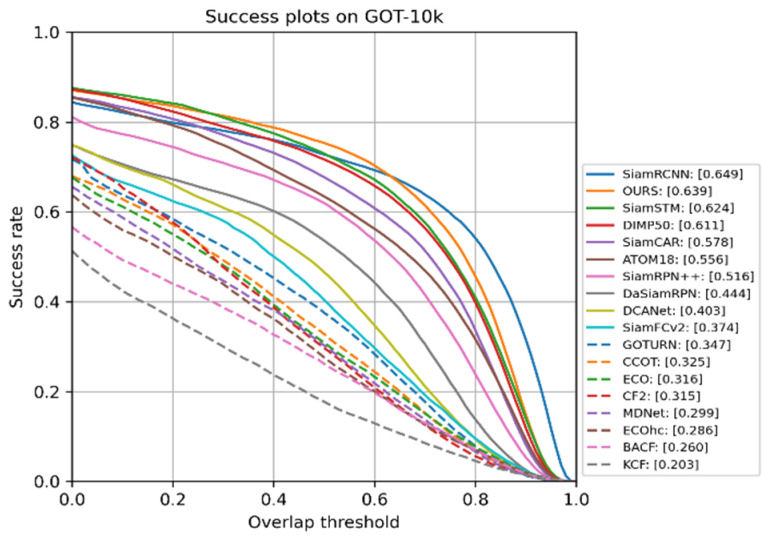
Comparison with SOTA trackers on GOT-10k, with success rate.

GOT-10k is a large-scale benchmark including several common challenges in object tracking, including occlusion, scale variation, aspect ratio variation, fast motion, illumination variation, and small/large objects. The dataset contains more than 10,000 video segments of real-world moving objects and over 1.5 million manually labeled bounding boxes. The dataset is backboned by WordNet, and it covers a majority of 560+ classes of real-world moving objects and 80+ classes of motion patterns. Success rate is the ratio of the frames whose overlap between ground truth and prediction bounding box is over 50% to total frames. As shown in Figure 6, our CTT surpasses DiMP50 and SiamRPN++ by 2.8 and 12.3 points in SR (success rate), respectively. Although our method lagged behind SiamRCNN [46] by 1% in accuracy, we achieved a speed of 28FPS, which is much faster than 2FPS by 14 times.

#### 4.2.2. VOT2019

VOT2019 and VOT2018 [47] both contain 60 videos, replacing 12 simple scenes in VOT2018 with more complex ones, namely VOT2019. So, we directly evaluated CTT on the 2019 version. Tracking performance metrics are determined by accuracy, robustness, and EAO. Accuracy is the intersection of union between the predicted bounding box and ground truth in each frame, then we can get the average accuracy of a single video, while robustness is considered as accuracy with the least correlation. Specifically, it is to calculate the number of the tracker fails of a single video, which means that the intersection of union between the predicted bounding box and ground truth is 0. Instead of using accuracy and robustness directly, EAO is a comprehensive evaluation metric that is associated with both accuracy and robustness. Our CTT has a real-time speed at 57FPS on VOT2019. As illustrated in Table 1, accuracy is similar to the other methods, however, robustness and EAO are relatively lower than the TrTr. There are two reasons why our proposed CTT performs worse than other trackers on VOT2019. First, we observe that when the small object has fast motion (common on VOT2019), the predicted bounding box is always hard to keep up with the ground truth, that is why our model has a lower robustness and EAO. In visual target tracking, for the sake of tracking the small object, the FPN is always introduced to the network such as SiamRPN++. However, using the FPN in transformer can greatly affect the real-time performance of the network, so we do not employ the FPN under comprehensive considering about speed and EAO. Second, some trackers, such as TrTr, will adjust hyperparameters separately for each test set, such as search size, window factor of cosine window, etc. We think that adjusting hyperparameters should not be the focus of our work, so we apply the same parameters to all test sets, which may cause our CTT to perform poorly on some test sets.

#### 4.2.3. OTB-100

In case of tracking failure, except that VOT has a restart mechanism, other test sets follow the one-pass evaluation principle, that is, reinitialization is not allowed. We evaluate on the OTB-100 benchmark which contains 100 sequences. OTB-100 has many challenging scenes in visual tracking, such as illumination variation, scale variation, occlusion, deformation, motion blur, fast motion, in-plane rotation, out-of-plane rotation, out-of-view, background clutters, and low resolution. Precision is the percentage of video frames whose distance between the center points of the bounding box estimated by the tracker and the center of the ground-truth is less than a given threshold. Success is the same as GOT-10k’s success rate. We compared the CTT with the recent excellent trackers in precision and success. The detailed results can be seen in Table 2. Our CTT marks the third best while running with 28FPS. The performances of our CTT on OTB-100 and VOT2019 are not excellent enough. Both OTB-100 and VOT2019 have several fast motion sequences, but this is not the main reason why our network is far less effective than TrTr on OTB-100. When we retest TrTr in our configuration with the same as CTT, we find that TrTr will reset the tracker when the prediction bounding box near boundary, which is contrary to the principle of one-pass evaluation.

#### 4.2.4. NfS and UAV123

NfS means Need for Speed which is a benchmark for visual object tracking. NfS dataset consists of 100 videos from real world scenarios, and the version we evaluated is about 30FPS. All frames are annotated with axis aligned bounding boxes, and all sequences are manually labeled with nine visual attributes, such as occlusion, fast motion, background clutter, etc. The videos of UAV123 were captured from low-altitude UAVs with sequences from an aerial viewpoint. It contains a total of real-time video of 123 UAV applications and more than 110K frames. The evaluation criteria of tracker performance of NfS is accuracy, which has the same meaning in VOT2019. Following the evaluation strategy of OTB-100, all trackers are compared using two measures: precision and success in UAV123.

We compared our CTT with the top-performing trackers with accuracy on NfS and UAV123. Our method achieves the best overall performance with the highest accuracy compared to previous methods on the NfS, and obtains the second place on the UAV123. We show the results in Table 3. Our tracker’s speed of NfS and UAV123 are 56 and 34 frames per second.

#### 4.2.5. LaSOT

**Figure 7:**
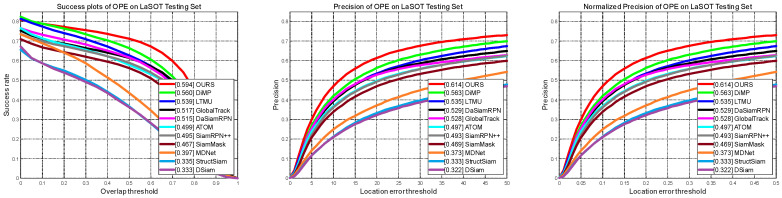
Comparison with SOTA trackers on Success, Precision, and Normalized Precision on LaSOT.

LaSOT is a high-quality benchmark for large-scale single object tracking. LaSOT consists of 1400 sequences with more than 3.5 million frames in total, making LaSOT the second largest densely annotated tracking benchmark. The average video length of LaSOT is more than 2500 frames, and each sequence comprises various challenges deriving from the wild where target objects may disappear and reappear again in the view. LaSOT is divided into training and test subsets according to 4:1. That is, for each category, we select 16 videos for training and 4 videos for testing, and finally, we can obtain 1120 training videos and 280 test videos. We calculate normalized precision and success (rate) by one-pass evaluation, which are the same as OTB-100. As shown in Figure 7, comparing with ten comparable trackers, our tracker achieves top performance in terms of success, precision, and normalized precision at about 50FPS.

#### 4.2.6. TrackingNet

TrackingNet is a large-scale target tracking dataset and benchmark. Typical challenge scenarios include small targets, occlusion, fast motions, and similar interference in background, etc., in the wild. It provides more than 30K videos with more than 14 million dense bounding box annotations. In addition, TrackingNet introduces a new benchmark composed of 500 novel videos. The calculation of accuracy and normalized precision is no different from LaSOT. The results, shown in Table 4, of our proposed model obtains 0.772 of AUC and 0.829 of normalized precision which ranks second. However, similar results have been seen with GOT-10k; due to the complex network structure of SiamRCNN, we are not optimistic about its real-time speed.

#### 4.2.7. Real-time Speed

At the end of this section, in order to highlight the real-time speed of our CTT, we compare the speed with SiamBAN and TrTr. It is worth noting that our experimental environment is completely consistent with CTT. As shown in Table 5, our CTT performs better than transformer architecture TrTr.

## 5. Discussion

In this section, we perform an ablation study of our proposed transformer architecture for object tracking on NfS. Then, we demonstrate the comparison of our proposed CTT with SiamBAN and TrTr (online) by visible results.

### 5.1. Ablation Study

In this section, we perform a comprehensive analysis of our proposed transformer architecture for object tracking on NfS.

#### 5.1.1. The Spatial and Channel Attention

In the original visual object tracking network based on Siamese networks, the structure of the target branch is shared with that of the search branch. However, the search image may contain many objects except the target. These objects and the background can be regarded as negative samples. In other words, the ratio of area occupied by positive and negative samples may be relatively low in visual object tracking.

According to the rules of the control variable method, we evaluate the effect of adding attention to the target branch on accuracy and precision of NfS test dataset during training and referencing, and compare the performance of adding SA, CA, SA&CA, and no attention to the target branch, respectively. As can be seen from Table 6, accuracy and precision, without adding any attention to the target, increased by 0.2 points, 0.4 points, 1.9 points, and 3.4 points, respectively, compared with adding SA or SA to the target branch. The accuracy and precision increase and decrease by 0.4 and 0.1 points, respectively, when no attention is added to the target compared to adding SA&SA to the target branch. We believe that if only one of SA or CA is added, neurons cannot learn the target information well. The reason for the results in Table 6 is that if we pay too much attention to the dimension of feature channel information of the target branch, its spatial dimension information may be relatively weakened; this problem is mitigated when both SA and CA are added to the target branch in parallel.

Features extracted by the target branch are matched with features extracted by the search branch in Siamese networks. The search image may contain many objects other than the target, which leads to a decrease in the robustness of the model if we encounter interfering objects. Therefore, we should pay more attention to the object rather than the negative samples so that the transformer can obtain more accurate target features before the stage of feature fusing. Thus, this particular “attention” operation can help the self-attention mechanism to emphasize the regions of the target object.

#### 5.1.2. Skip Connections

It can be seen from Table 6 that training with SA&CA has the best performance. On the basis of keeping SA&CA unchanged, the results of the ablation study with the skip connections are shown in Table 7. We compare the two tracking performances, whether the encoder layers with or without skip connections are used to train the model. Accuracy and precision increase by 0.2 and 0.1 points over no skip connections in NfS. In our tracking network as shown in Figure 2, the inputs of transformer’s encoder are extracted from the Resnet-50’s layer 3. We assume that the stacked self-attention transformer needs to calculate encoder-decoder attention values between feature embeddings from the backbone for several times, which may make the network overfitting, and lead to reducing the robustness to unknown tracking objects. Therefore, we add skip connections between the encoders and decoders of the target branch and the search branch, respectively. In the case of no increase in network parameters, skip connections can keep original information between deep encoders, and it is not easy to gradient vanishing during training, which is conducive to fast convergence of deep transformer and strengthening the stability of our model.

#### 5.1.3. The Number of Encoders and Decoders

**Figure 8:**
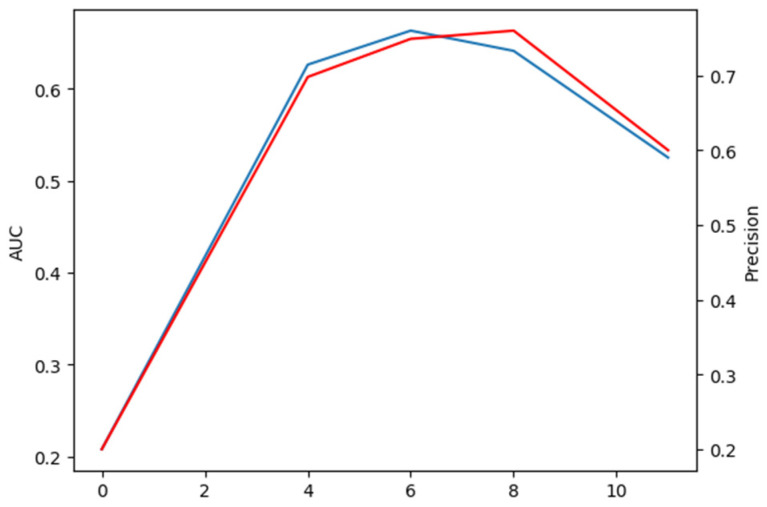
Analysis of the impact of numbers of encoders and decoders in transformer architecture of Figure 2. AUC and precision as shown by red and blue line, respectively.

It can be observed from Table 7 that the skip connections can improve the accuracy and precision of our CTT. We train models with the number of encoders and decoders with different combinations on the consumption of keeping the skip connections unchanged; the extensive comparison results are shown in Figure 8. The relative number of encoders and decoders has a significant impact on our transformer model. It is worth considering that for our model to work best, the number of encoders and decoders is not as close as possible, unlike models in ViT and other visual transformers. There are two reasons as follows:

1. The parameters of the encoder layer redesigned by us are less than those of the decoder layer. Therefore, when designing the number of encoders and decoders, it is reasonable for the number of encoders, as more than the number of decoders, to keep balance of total parameters of encoders and decoders;
2. The main task of the encoder is to carry out global information interaction between features obtained from the backbone, and it is the key to solve the problem of the tracking process being susceptible to similar target interference. The decoder is responsible for decoding the information already fused in the encoder and sending it to the head module, therefore, the decoder is also essential.

An interesting phenomenon is that if we compare the encoders only with the decoders only in transformer, we find that the encoders only perform much better on the test set than the decoder only. Considering the data in Figure 8, we can infer that the model is underfitted when using encoders only, because there are not enough parameters to fit our tracking model. When using decoders only, the model can be very badly over-fitted, resulting in poor performance on the test set.

Since the number of encoders is even, we focus on the comparison of encoder and decoder numbers 6, 5, and 8, 3, respectively. The AUC and precision of the former case are 2.2 points higher and 1.1 points lower than those of the latter case, respectively. In summary, when the number of encoders and decoders are 6 and 5 respectively, our CTT performs best at accuracy and robustness.

### 5.2. Qualitative Evaluation

**Figure 9:**
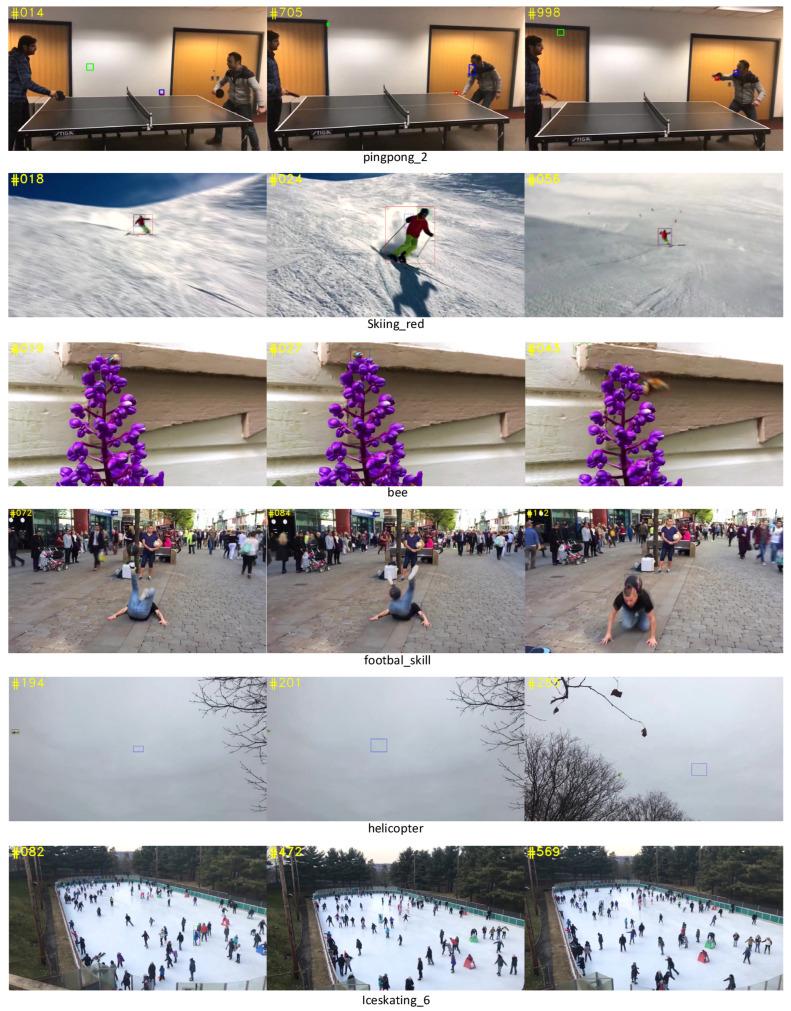
A comparison of CTT (red) with SOTA trackers SiamBAN (green) and TrTr (online) (blue) on NfS.

With the above ablation studies, we have proved the effectiveness of the components of our proposed model. To further verify it, we demonstrate the comparison of our proposed CTT with SiamBAN and TrTr (online) in the case of the presence of similar looking objects (distractors), multi-scale transform, blur, small object tracking, and rapid posture changes with occlusion. The prediction boxes for CTT, SiamBAN, and TrTr are shown in red, green, and blue in Figure 9.

#### 5.2.1. Similar Objects

In the long sequence pingpong_2, we pick three representative frames. At frame 14, it is obvious that SiamBAN’s prediction box based on traditional CNN can be easily transferred to the wall, as the ping-pong ball and the wall are both white. TrTr and CTT based on transformer’s architecture track the ping-pong ball accurately. At frame 705, when the ping-pong ball approaches the white line on the table, it can be observed that the CTT’s box precisely keeps up with the ball, while TrTr keeps track of the golden door handle. The same phenomenon occurs in the sequence footbal_skill, frame 84, SiamBAN, and TrTr which are disturbed by dark clothes on another person and their own black clothes, respectively. Note that SiamBAN and CTT are offline trackers, while TrTr is an online updated tracker. We observe that online update tracker based on transformer is not always better than us when we encounter similar object interference. Although TrTr can update the target branch, its prediction box will closely follow the interfering object if the tracker meets similar interference for a long time, because its classification result depends on the weighted sum of offline classifier and online classifier, and the specific weighted ratio is a hyperparameter, which is difficult to adjust.

#### 5.2.2. Multi-Scale Transform

In the short sequence Skiing_red, it is clear from frame 18 or 24 that SiamBAN and TrTr do not cope well with rapid multi-scale changes of the skier.

In our proposed network, Q, K, and V in the target branch and the search branch are not directly input into the encoders, but mixed and then crossed into different encoders so that the target information can fully interact with the search information. In this way, our model can find the target in the search branch in time to deal with the multi-scale changes.

#### 5.2.3. Blur

The sequence bee well restores a common blur scene in daily life. When the motion pattern of the bee to be tracked is blur, and the color of the bee is similar to the wall, as shown in frame 43 of the sequence bee in Figure 9, CTT is not easily disturbed as SiamBAN and TrTr because the feature fusion module of our network is designed more reasonably.

#### 5.2.4. Small Object Tracking

As shown in the tracking results in Figure 9, in the face of a challenge such as accurate tracking of the small moving object, both CTT and SiamBAN can complete the task, but our tracking result is more accurate, while TrTr is completely obsessed with tracking the “background”.

#### 5.2.5. Rapid Posture Changes with Occlusion

As shown in Figure 9, the tracking results in Iceskating_6 sequence show that the robustness of the three trackers are very excellent when tracking the object with rapid posture changes and partial occlusion. In terms of accuracy, SiamBAN performs worst, while CTT and TrTr have a very well result approximately.

## 6. Conclusions

In this paper, we propose an end-to-end training and inferencing tracker. We replace the FPN and cross-correlation operation used in common Siamese networks with an encoder-decoder transformer architecture. Our proposed CTT is mainly to solve the interference problem of similar objects in visual object tracking. Therefore, unlike the previous visual transformer (ViT, DETR, TrTr…), our trasnformer architecture does not input the features passing through the backbone directly and sequentially into the encoder-decoder structure, but rearranges the features in the target branch and search branch, and inputs them into the encoders and decoders. In addition, we add the SA&CA to the target branch to further improve the robust tracking performance of target location. Moreover, the simplified encoders and skip connections cause our model to obtain a better real-time speed. Our proposed off-line model tracker achieved performance comparable to the SOTA online update model in most benchmarks, especially dealing with the similar objects, but there is still room for improvement on datasets VOT-2019 and OTB-100.

In future work, we will expand transformer architecture to solve the challenge of fast motion by utilizing the relationship between upper and lower frames while maintaining the real-time inference speed.

## Figures and Tables

**Table 1:** Comparison with SOTA trackers on VOT2019, with accuracy (A), robustness (R), and expected average overlap (EAO).

Tracker	A↑	R↓	EAO↑
ATOM [48]	0.603	0.411	0.292
SiamRPN++	0.599	0.482	0.285
SiamBAN	0.602	0.396	0.327
DiMP50 [49]	0.594	0.278	0.379
Ocean [50]	0.594	0.316	0.350
MAML-T [51]	0.570	0.366	0.313
TrTr-offline	0.608	0.441	0.313
TrTr-online	0.609	0.326	0.317
OURS	0.595	0.411	0.299

**Table 2:** Comparison with SOTA trackers on OTB-100, with Precision (P) and Success rate (SR).

Tracker	P↑	SR↑
SiamRPN++	0.915	0.696
DiMP50	0.899	0.686
MDNet [52]	0.909	0.678
ATOM	0.879	0.667
SiamBAN	0.897	0.685
TrTr-offline	0.919	0.702
TrTr-online	0.933	0.711
DaSiamRPN [53]	0.880	0.658
OURS	0.904	0.687

**Table 3:** Comparison with SOTA trackers on NfS and UAV123, with Accuracy (A).

Tracker	A of NfS↑	A of UAV123↑
MDNet	0.422	0.528
ATOM	0.548	0.658
DiMP50	0.620	0.653
SiamBAN	0.594	0.631
SiamRCNN	0.639	0.649
PrDiMP50 [54]	0.635	0.680
TrTr-offline	0.552	0.611
TrTr-online	0.631	0.616
OURS	0.641	0.667

**Table 4:** Comparison with SOTA trackers on TrackingNet, with Accuracy (A) and Normal Precision (N-P).

Tracker	A↑	N-P↑
ATOM	0.703	0.771
SiamRPN++	0.733	0.800
DiMP50	0.740	0.801
SiamAttn	0.752	0.817
PrDIMP50	0.758	0.816
SiamRCNN	0.812	0.854
TrTr-offline	0.693	0.772
TrTr-online	0.710	0.803
OURS	0.772	0.829

**Table 5:** Comparison with SiamBAN and TrTr on VOT2019, OTB-100, and UAV123 real-time speed (FPS).

Tracker	VOT2019	OTB-100	UAV123
SiamBAN	39	46	45
TrTr-offline	29	26	28
TrTr-online	27	27	27
OURS	57	28	34

**Table 6:** Analysis of the impact of SA and CA in backbone’s target branch of Figure 3 which is evaluated on NfS dataset.

	A↑	P↑
No SA or CA	0.637	0.761
SA	0.635	0.757
CA	0.618	0.727
SA&CA	0.641	0.760

**Table 7:** Analysis of the impact of skip connections in Transformer architecture of Figure 2 evaluated on NfS dataset.

	A↑	P↑
No skip connections	0.641	0.760
Skip connections	0.643	0.761

## Data Availability

Not applicable.
